# EEG dynamics reveal a dissociation between storage and selective attention within working memory

**DOI:** 10.1038/s41598-019-49577-0

**Published:** 2019-09-18

**Authors:** Eren Günseli, Johannes Jacobus Fahrenfort, Dirk van Moorselaar, Konstantinos Christos Daoultzis, Martijn Meeter, Christian N. L. Olivers

**Affiliations:** 1 Columbia University, Department of Psychology, New York, USA; 2 Vrije Universiteit Amsterdam, Department of Experimental and Applied Psychology, Amsterdam, Netherlands; 3 Institute for Brain and Behavior Amsterdam, Amsterdam, Netherlands; 4 Panteion University, Department of Psychology, Athens, Greece; 5 Vrije Universiteit Amsterdam, LEARN! Research Institute, Amsterdam, Netherlands

**Keywords:** Attention, Working memory, Human behaviour

## Abstract

Selective attention plays a prominent role in prioritizing information in working memory (WM), improving performance for attended representations. However, it remains unclear whether unattended WM representations suffer from information loss. Here we tested the hypothesis that within WM, selectively attending to an item and stopping storing other items are independent mechanisms. We recorded EEG while participants performed a WM recall task in which the item most likely to be tested was cued retrospectively during retention. By manipulating retro-cue reliability (i.e., the ratio of valid to invalid cue trials), we varied the incentive to retain non-cued items. Storage and selective attention in WM were measured during the retention interval by contralateral delay activity (CDA) and contralateral alpha power suppression, respectively. Soon after highly reliable cues, the cued item was attended, and non-cued items suffered information loss. However, for less reliable cues, initially the cued item was attended, but unattended items were kept in WM. Later during the delay, previously unattended items suffered information loss despite now attention being reallocated to their locations, presumably to strengthen their weakening traces. These results show that storage and attention in WM are distinct processes that can behave differently depending on the relative importance of representations.

## Introduction

Working memory (WM) is essential to storing and manipulating information online for a variety of cognitive tasks^1–4^. However, its capacity is limited^5,6^ and thus only the most task-relevant information should be selected for storage in WM^7,8^. Attention is the mechanism by which most task-relevant representations are prioritized. There is now a large body of evidence showing that attention and WM are heavily intertwined^9–12^, such that attention may be crucial for maintaining an item in WM successfully^13–21^. However, more recently alternative theoretical frameworks have been proposed that argue storage of an item in WM should be dissociated from prioritization of (i.e., attending to) that item^22–27^. Thus, there is no consensus yet on the relationship between WM and attention.

Much of the evidence for a central role of selective attention in WM storage comes from studies using retrospective cues. Such “retro-cues” indicate which of the memory representations is most likely to be tested and therefore is the most task-relevant. Importantly, retro-cues are presented after memory encoding. Thus, they act on stored WM representations rather than on encoding of stimuli. Nevertheless, retro-cues have been suggested to result in the attentional selection of the cued representation within WM in a similar way as attentional selection operates during perception, relying on highly overlapping neural networks^28–30^. Attentional selection, in turn, has been claimed to improve storage and increase the accessibility of the cued item within WM^25,31,32^. The behavioral consequence is better memory performance for the attended representation compared to a ‘no-cue’ or neutral condition where all items are presumably equally attended^33–35^.

The evidence for improved memory performance for attended representations does not in itself indicate that attention is *necessary* for storing that representation. Proving that attention is essential for storage requires showing that items *suffer* when attention is cued elsewhere relative to when attention is directed equally to all items. However, so far, the fate of unattended WM representations has been unclear. Memory performance for unattended representations can be tested by probing a non-cued representation on a minority of the trials. Lower memory performance on these invalid cue trials compared to neutral or no-cue trials is referred to as an ‘invalid cueing cost’. Such invalid cueing costs have indeed been found in some studies, and have been taken as evidence that attention is necessary for WM storage^36–38^. However, using very similar cueing procedures, some other studies did not find an invalid cueing costs^25,32,40^, suggesting a dissociation between storage and selection.

As we have proposed earlier^39^, the fate of unattended representations might depend on their perceived future relevance. In retro-cue studies, perceived future relevance would be inferred from the reliability of the retro-cue (i.e., the proportion of valid to invalid retro-cue trials). Typically, studies that failed to observe invalid cueing costs used lower retro-cue reliabilities^25,40,41^ than studies that found invalid cueing costs^31,36,38,42^. In line with this, in a behavioral study, we observed invalid cueing costs only when the retro-cue had high reliability (i.e., 80% valid). There was no invalid cueing cost for a less reliable but still above-chance retro-cue (i.e., 50% valid, with the chance level being at 25% in both conditions). While the presence of invalid retro-cue costs varied with retro-cue reliability, benefits of *valid* retro-cues were present in both conditions, though they were larger for 80% valid cues^39^. These results can explain the discrepant findings in the literature if we assume that attending to an item in WM can be dissociated from the decision to either continue or cease storage of remaining items. For both moderately and highly reliable cues, it is beneficial to attend to the cued item, as it is more likely to be tested than non-cued items. However, only for highly reliable cues, it is also worth dropping the non-cued items from memory, while for moderately reliable cues it is worth holding on to the non-cued items.

Our behavioral work provides initial evidence for the idea that attention to versus storage of an item should be dissociated when interpreting the effects of retro-cueing. However, there is an alternative scenario that can explain the reliability effects on performance for non-cued items. Increasing the retro-cue reliability might result in more attention to the selected representation without affecting the probability with which the unattended representations are dropped from WM. Under this scenario, items remain stored in WM regardless of cue reliability, but they become more vulnerable to interference from the test display when unattended. The test display is in itself a stimulus that may overwrite a fragile memory representation, particularly when they are unattended^43–46^. Perceptual interference would then result in larger invalid cueing costs for highly reliable cues even if unattended items were still stored until the test display. Thus, the ‘protection against interference’ account explains larger invalid cueing costs for more reliable cues due to perceptual interference at test as opposed to differential levels of storage during the retention interval. On the other hand, our aforementioned ‘attention-storage dissociation’ account predicts that unattended items can be dropped during the retention interval depending on their perceived future relevance. Our behavioral measure is blind to the underlying mechanisms during retention and therefore cannot differentiate between these scenarios. Thus, developing a complete understanding of the relationship between attention and storage is difficult by using behavioral measures alone.

**Figure 1:**
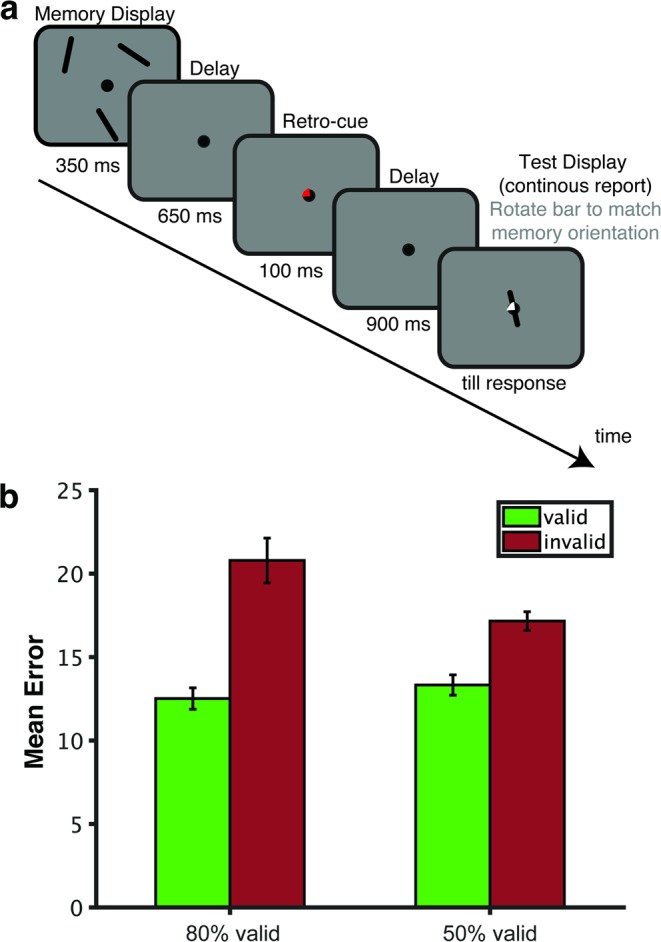
(**a**) Retro-cue experimental procedure. Participants were asked to remember the three orientations shown in the memory display. After a blank interval, a retro-cue was presented pointing to the location of the item (in this example top-left) that was most likely to be tested. Retro-cues were not always valid. Following a second blank interval, the test display was presented during which participants were asked to rotate a randomly-oriented bar to match the orientation of the tested item (which in this example is the item presented on top-left; hence the retro-cue was valid). (**b**) Average error for reporting the probed orientation in each condition. Valid and invalid trials are shown in green and red respectively. Error bars represent standard errors of the mean for normalized data, i.e., corrected for between-subjects variance (Cousineau, 2005). Retro-cue validity effect (difference in error between invalid vs. valid cue trials) was larger for highly reliable cues than less reliable cues.

To more directly investigate if and how retro-cue reliability affects attention and storage in WM during *retention* before the test display, we used EEG recordings to measure these processes in a time-resolved manner during the memory delay interval. The experimental procedure is shown in Fig. 1a. We used a continuous report WM task to obtain a sensitive measure of memory performance. The memory display contained three line segments of different orientations, one on the vertical midline, and the other two presented left and right from fixation. After a blank interval, a retro-cue indicated which of the memory representations was most likely to be tested by retrospectively pointing to its location in the memory display. Only lateral cue trials were included for the EEG analysis since both of our EEG indices of interest (see below) require a lateral asymmetry in the location of the attended and stored item. Critically, to vary the incentive to retain non-cued items, we manipulated retro-cue reliability (i.e., the proportion of valid to invalid trials) across blocks: The cue was 50% valid in half the number of blocks, and 80% valid in the other half.

As a proxy for *attention* being directed within WM we used contralateral power suppression in the alpha band (8–14 Hz). Alpha power over the parietal-occipital electrodes on the hemisphere contralateral to the attended item has been found to be reduced relative to the ipsilateral electrodes, both during perception and after perception, within WM^47–52^. We hypothesized that if the cued item is attended more during storage for highly reliable cues, then we should observe a larger contralateral alpha suppression for highly reliable retro-cues than for cues of lower reliability. As a marker for *storage*, we focused on the CDA, which is a sustained negativity over the parietal-occipital electrodes on the hemisphere contralateral to remembered stimuli. It has been observed to be sensitive to the visual WM load, and converging evidence suggests that it is an index of visual WM storage^53–56^. We reasoned that if non-cued representations are dropped following a retro-cue at least on a proportion of trials, then a CDA should emerge contralateral to the attended item, since dropping an item on one side results in an imbalance in the number of items stored in each hemisphere^57^. If, as we hypothesized, the likelihood of dropping an item depends on retro-cue reliability, we should see a stronger CDA emerge in the high-reliability condition than in the low-reliability condition. Alternatively, if retro-cue reliability has no effect on storage, we should see no differential CDA, and only find attentional effects as expressed through alpha suppression. Any dissociation between lateralized alpha and CDA would provide further evidence for independent attention and storage mechanisms within working memory.

## Method

Thirty-two healthy volunteer university students (ages 18–35) participated in the experiment for course credit or monetary compensation. Two participants were excluded; one due to excessive noise in their EEG recordings and one due to poor behavioral performance (see Data Analyses), leaving 30 participants (22 female) of whom the data was analyzed. The study was conducted in accordance with the Declaration of Helsinki and was approved by the Scientific and Ethical Review Board (Dutch abbreviation: VCWE) of the Faculty of Behavioral and Movement Sciences. Written informed consent was obtained prior to the experiment. Datasets are available online on Open Science Framework at https://osf.io/bgpxc/?view_only=3b8dd8f9e4fa42d68ac84db90f76e25d.

The procedure is shown in Fig. 1. Each trial started with the presentation of the fixation circle of radius 0.33°, for a duration jittered between 1200–1600 ms. Then, the memory display was presented for 350 ms. It consisted of three black oriented bars (2.08° × 0.25° visual angle) located at 60 (top right), 180 (bottom) and 300 (top left) degrees relative to the top of an imaginary circle of radius 3.50°. We used a memory load of three items in order to tax WM without contaminating measurements with non-encoded items. The orientation of each bar was chosen at random with the restriction that bars within the same trial differed by at least 10°. The retro-cue was presented for 100 ms following a blank interval of 650 ms during which only the fixation circle was presented. The retro-cue was identical to the fixation circle except for one quarter (90°) was now filled with either red, 27.08 Cd/m^2^, or green, 24.10 Cd/m^2^, depending on the reliability condition (order counterbalanced). For the initial practice phase where the cue was 100% valid, the retro-cue fill color was orange (53.46 Cd/m^2^). Following the retro-cue, there was a blank interval of 900 ms in which only the fixation circle was presented. Then the test display was presented till response. It contained a probe cue pointing to the location of the tested representation and a randomly oriented probe bar that were both presented at the center of the screen. This probe cue was the same as the retro-cue except that the filling color was white. Participants were asked to indicate the orientation of the bar at the tested location as precise as possible by rotating the probe bar using the mouse and pressing the left mouse button. After a mouse response was made, the correct orientation was indicated by a central white bar for 100 ms. The screen was empty during the inter-trial interval which was jittered between 1200–1600 ms.

The retro-cue was 80% valid for half of the experiment and 50% valid for the other half (order counterbalanced). There were 10 blocks of 50 trials. Each validity condition (i.e., valid and invalid) was randomly intermixed within each block. Before each reliability condition, participants were informed about the validity ratio of the retro-cue and they performed a practice session of 25 trials to get used to this particular validity ratio. At the beginning of the experiment, there was an initial practice session of 25 trials with a 100% valid cue to make participants familiar with using the experimental procedure. At the end of each block, participants received feedback on block average and grand average error (i.e., the difference between the original tested orientation and the responded orientation).

### EEG data acquisition

The electroencephalogram (EEG) and electro-oculogram (EOG) were recorded from 70 sintered –AG/AgCl electrodes positioned at 64 standard International 10/20 System sites and 6 external locations mentioned below, using the Biosemi ActiveTwo system (Biosemi, Amsterdam, the Netherlands). We did not perform impedance measurements as recommended by Biosemi: High impedance has been suggested to have a minor impact on data quality in cool and dry environments^58^. The vertical EOG (VEOG) was recorded from electrodes located 2 cm above and below the right eye, and the horizontal EOG (HEOG) was recorded from electrodes 1 cm lateral to the external canthi. The VEOG was used in the detection of blink artifacts, and the HEOG was used in the detection of horizontal eye movement artifacts. Electrophysiological signals were digitized at 512 Hz.

### Data analysis

#### Behavior

Error scores on the memory test were calculated as the difference between the original orientation of the tested memory bar and the orientation of the response. One participant with an average absolute error value higher than 2.5 standard deviations above the grand average of the group was excluded from the analysis. Absolute error for the tested item was entered into a repeated-measures ANOVAs with the within-subjects factors of retro-cue reliability (80% valid; 50% valid) and retro-cue validity (valid; invalid).

We also estimated the guess rate (i.e., reporting a random orientation), swap rate (i.e., reporting a non-tested representation), and sd (inverse of precision) based on the width of the response distribution around the target using MemToolbox (memtoolbox.org)^59^. To test if the large trial number imbalance between valid and invalid trials in 80% valid condition (200 vs. 50) has an impact of parameter estimates, we took 200 trials in 80% valid condition and downsampled 50, 100, and 150 trials over 20 iterations. We found that having a lower number of trials significantly inflates guess rate and swap rate estimates, but more importantly, increases the variability of each estimate (i.e., decreases their reliability). Thus, we report only raw errors and not the parameter estimates from the swap model.

#### EEG analysis: General

Only lateral cue trials were included for the EEG analysis since both of our EEG indices of interest require a lateral asymmetry in the location of the attended or stored item. All EEG analyses were carried out using the EEGLAB toolbox^60^ and custom scripts implemented in MATLAB (The MathWorks, Inc., Natick, MA). Due to unknown reasons, three participants had parts of EEG data missing (10, 11 and 26 trials). Noisy electrodes were interpolated using the “eeg_interp.m” function of EEGLAB with the spherical interpolation method, which resulted in the interpolation of three electrodes each for two participants (FC2, C6, PO3; CP3, PO3, P4). None of these electrodes were used in the statistical analysis. Trials with recording artifacts (muscle noise and slow drifts) and ocular artifacts (blinks and eye movements) were rejected manually by visually inspecting the EEG and EOG electrodes respectively. Artifact rejection was performed in the absence of any knowledge about the conditions. Individuals were excluded from analyses if, after all the artifact rejections, the remaining number of trials per condition was lower than 80 trials. This led to the rejection of one participant. For the remaining participants, on average 9.8% of all trials were rejected due to artifacts, leaving on average 139 and 141 lateral cue trials for analyses (with a minimum of 105 and 111 trials), for 80% valid and 50% valid blocks respectively.

#### ERP analysis: CDA

ERPs were computed with respect to a 200 ms pre-stimulus baseline period, between −500 to 1500 ms around the retro-cue display and were re-referenced offline to the average of left and right mastoids. The data were band-pass filtered between 0.01 Hz and 6 Hz using a two-way IIR Butterworth filter of 4^th^ order as implemented by the ft_preproc_bandpassfilter.m function of FieldTrip Toolbox. We chose an upper limit of 6 Hz to remove the alpha-band activity from the CDA calculation to fully isolate the two signals. However, our main findings (i.e., CDA being significant only for 80% valid condition early in the trial, but for both 50% and 80% valid conditions late in the trial) were identical when the CDA was calculated using a bandpass filter of 0.01–40 Hz. Signal was resampled at 500 Hz using “pop_resample.m” function of EEGLAB.

The CDA was calculated as the difference waves between electrode sites contralateral versus ipsilateral to the location of the retro-cued item. Previous studies measuring the CDA have typically found maximal values at posterior/occipital electrodes and started measuring the CDA at ~300–400 ms from the onset of the memory display following the N2pc (~200–300 ms) which signals individuation of selected items^53,61,62^. Based on these studies and visual inspection of the topographic distribution of lateralized voltage in posterior/occipital regions we calculated the CDA at P7/8, PO7/8, and O1/2 as contralateral minus ipsilateral to the retro-cued location starting from 400 ms following the retro-cue onset till the onset of the test display (i.e. 900 ms after cue onset). The CDA averaged across electrode pairs and times of interest was entered into a repeated-measures ANOVA with the within-subjects factor of retro-cue reliability (80% valid; 50% valid). Average CDA values were also tested against zero using one-sample t-tests. Additionally, given that CDA is a sustained negativity at electrodes contralateral to items stored in WM, we hypothesized that if the reliability effect on CDA reflects a boost for the representation of the cued item, then it should be evident in the signal contralateral to the *cued* item, while if it reflects dropping of the non-cued item then the reliability effect should be observed in the signal contralateral to the *non-cued* item (i.e., ipsilateral to the cued item). To test this, the average contralateral and ipsilateral signals were entered into a repeated-measures ANOVA with within-subjects factors of laterality (contralateral; ipsilateral to the cued item) and retro-cue reliability (80% valid; 50% valid).

In order to investigate the dynamic time course of the reliability effect, the CDA at each time point was tested against zero and also compared across reliability conditions at a group level using cluster-based permutation testing by estimating the permutation *p*-value using a Monte Carlo randomization procedure^63^. For this analysis, we randomly shuffled the condition labels (e.g., 50% valid vs. 80% valid) 1000 times to approximate the null distribution of the t statistic. Multiple comparisons correction was established using cluster-based permutation testing^63^. First, four or more temporally adjacent data points with a p-value smaller than 0.05, as revealed by a t-test (two-tailed), were clustered together. Then, a cluster-level statistic was calculated by taking the sum of the t-values within each cluster, separately for positive and negative clusters. The p-value for each cluster was calculated as the proportion of times the largest summed absolute t-value under random permutation exceeds the sum of the absolute t-values within the observed cluster. A cluster was considered significant if the calculated p-value was smaller than 0.05. Note that there are two separate p-value calculations described above as part of cluster-based permutation testing. First, to determine significant time points that form the basis of the clusters, second to determine which of these clusters are significant.

#### Alpha band (8–14 Hz) power

Power analysis was performed using the same trials from the CDA analysis. We used custom-written MATLAB code to implement Morlet wavelet convolution. First, 19 linearly spaced complex Morlet wavelets ranging from 4 to 40 Hz (steps of 2 Hz) were created by multiplying perfect sine waves (e^i2πft^ where i is the complex operator, f is frequency, and t is time) with a Gaussian (${{\rm{e}}}^{-{{\rm{t}}}^{2}/2{{\rm{s}}}^{{\rm{2}}}}$, where s is the width of the Gaussian). The width of the Gaussian was set according to s = δ/2π*f* where δ represents the number of cycles of each wavelet, logarithmically spaced between 3 and 12 to have a good tradeoff between temporal and frequency precision^64^ (similar to our previous work^65^). The Fast Fourier Transform (FFT; i.e., frequency-domain convolution) was applied to both the EEG data and the Morlet wavelets, the outcome was multiplied in the frequency domain, and its inverse FFT was taken, resulting in a complex signal. We estimated the frequency specific total power at each time point by taking the square of the length of this complex signal (in Matlab: abs(X)^2^). Power data was baseline normalized separately for each condition (i.e. 50% valid left-cue, 50% valid right-cue; 80% valid left-cue; 80% valid right-cue) with decibel (dB) conversion according to 10 * log10(power/baseline), using −400 to −100 ms relative to the retro-cue onset as baseline^64^. The dB normalized data were averaged separately for contralateral and ipsilateral with respect to the side of the cued item at the electrode pairs of interest (P7/P8, PO7/PO8, O1/O2). Contralateral power suppression was calculated as the difference between contralateral vs. ipsilateral dB normalized power values. Based on previous work that show alpha-band power topography is sensitive to spatial selective attention^47–52^, we averaged the power values across the alpha-band (8–14 Hz). A whole frequency-range analysis showed that the effect retro-cue reliability was mainly restricted to our pre-defined frequency band of interest (see Supplementary Fig. 1).

Contralateral alpha band power averaged across electrodes and times of interest (400–900 ms, which is chosen to be the same time interval as the CDA analysis) were compared between 50% and 80% valid conditions using a repeated measures ANOVA. Average contralateral alpha band power values were also tested against zero using one-sample t-tests. Since we performed a reliability analysis separately for contralateral and ipsilateral signal for the CDA, for completeness we also performed it for lateral alpha power. Power values contralateral and ipsilateral power relative to the direction of the cued item were averaged across the time window of interest entered into a repeated-measures ANOVA with within-subjects factors of laterality (contralateral; ipsilateral to the cued item) and retro-cue reliability (80% valid; 50% valid). Lastly, contralateral power suppression at each time point was tested against zero and compared across reliability conditions at a group level with the same cluster-based permutation test as in the CDA analysis.

#### Investigating the effects of eye movements on EEG measures of interest

Eye movements can contaminate the EEG signal as a result of a potential difference between the cornea and fundus of the eye^66^. Even though we rejected trials with eye movements as reflected in HEOG electrodes, subtle differences in eye movements across retro-cue reliability conditions might have spuriously produced differences in CDA and lateralized alpha-band power. In order to evaluate this possibility, first we calculated the average potential difference between left and right HEOG electrodes during our time window of interest (i.e., 400–900 ms) separately for trials where the cued item was on the left vs. the right hemifield, and then averaged their absolute values. This allowed us to assess the magnitude of horizontal eye movements given that previous work has shown that an HEOG voltage difference of 16 µV corresponds to one visual degree of horizontal saccades^66^. Second, we compared this measure across 80% valid and 50% valid conditions using a repeated measures ANOVA to test if the magnitude of eye movements differed across reliability conditions. Third, to evaluate if the magnitude of eye movements might have driven our EEG measures of interest, we calculated correlations between average HEOG values and average CDA and contralateral alpha suppression values across individuals for both reliability conditions.

#### Correlation between EEG measures and behavior

To test if differences across reliability conditions in our EEG measures of interest predict behavior, we calculated the difference in invalid cueing cost (i.e., error in invalid trials minus error in valid trials) between 80% valid and 50% valid conditions, and correlated this with the CDA and contralateral alpha suppression differences between these two conditions. We calculated average CDA and contralateral alpha suppression at the time window in which the condition difference was significant within the retention interval as revealed by the aforementioned cluster-based permutation tests (400–514 ms for the CDA, and 700–834 ms and 852–900 ms the contralateral alpha suppression).

To test if variability in our EEG measures of interest predicts behavior, we calculated, per cell (participant; reliability condition; validity condition), Pearson’s correlations between trial-level CDA and error, and between trial-level contralateral alpha power and error (except invalid trials of 80% reliable condition, as there were too few trials in this condition). Then, average Pearson’s correlation coefficients were Fisher transformed to ensure normality and were tested against zero using one-sample t-tests.

#### Correlation between CDA and contralateral alpha suppression

In order to test whether selective attention within WM predicts storage in WM on a participant level, we performed Pearson correlations between the average CDA and the average contralateral alpha suppression across participants, separately for 50% valid and 80% valid blocks. In order to investigate the same relationship at a within-participant level, we calculated Pearson’s correlation coefficients across trials, per participant, per condition between our measures of interest (i.e., CDA and contralateral alpha suppression) averaged across the time-window of interest (i.e., 400–900 ms). Then, Pearson’s correlation coefficients per participant and per condition were Fisher transformed to ensure normality and were tested against zero separately for 50% valid and 80% valid blocks using one-sample t-tests. Given that the CDA and contralateral alpha suppression effects were specific to early and late time intervals respectively, we also performed this analysis separately for the time windows at which cluster-based permutation tests showed significant reliability effects on CDA (400–514 ms) and contralateral alpha suppression (700–834 ms and 852–900 ms).

## Results

### Behavior

Figure 1b shows mean absolute error (i.e. the absolute difference between the actual probed orientation and the responded orientation) for each condition. There was a main effect of validity on error, *F*(1, 29) = 27.75, *p* < 0.001, η_p_^2^ = 0.49. Errors were larger on invalid (*Mean* = 18.97, 95% CI [16.09, 21.84]) compared to valid cue trials (*Mean* = 12.92, 95% CI [11.62, 14.22]). Importantly, this validity effect (i.e. error on invalid trials minus the error on valid trials) was larger when the cue was 80% valid, *Mean* = 8.27, 95% CI [4.39, 12.15], compared to when it was 50% valid, *Mean* = 3.83, 95% CI [2.31, 5.35], as indicated by a validity × reliability interaction, *F*(1, 29) = 6.49, *p* = 0.016, η_p_^2^ = 0.18. There was no main effect of reliability on error, *F*(1, 29) = 2.41, *p* = 0.131, η_p_^2^ = 0.07. In sum, and as we expected, the effect of retro-cues on recall performance was larger for cues that were more reliable.

### Electrophysiology

#### Selective attention was allocated to the cued item independent of retro-cue reliability but was sustained only for highly reliable cues

**Figure 2:**
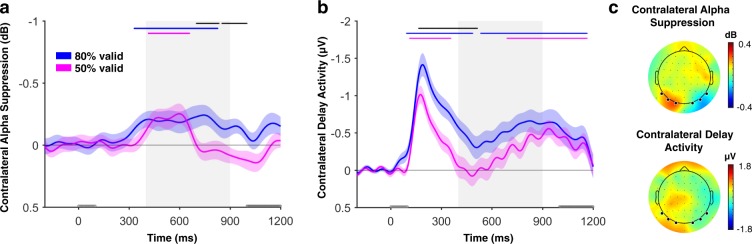
(**a**) Contralateral alpha suppression (CAS), and (**b**) contralateral delay activity (CDA) as indices of selective attention and storage in WM respectively, both time-locked to the onset of the retro-cue, are shown in different colors for 80% valid and 50% valid conditions. The gray area shows the time window of interest (400–900 ms). The gray rectangles on the x-axis show the timing of the retro-cue (0–100 ms) and the test display (from 1000 ms till response, which extends till 1200 ms on the plots). Markers along the top of each plot indicate the time points at which either the difference between the EEG measures in 80% valid and 50% valid conditions (black) or the EEG measure itself for each condition (blue for 80% valid and magenta for 50% valid) were significantly different than zero as determined by a cluster-based permutation test (*p* < 0.05; two-tailed). For highly reliable cues, the cued item was attended, and non-cued items were dropped from WM. For less reliable cues, non-cued items were initially unattended but were kept in WM until about the onset of the test display. This result suggests a dissociation between selective attention and storage within WM. Error bars represent standard error of the mean. (**c**) The scalp maps of CAS and CDA averaged across the time window of interest (400–900 ms after the cue onset) calculated as the difference of trials when the cued item was on the left minus when it was on the right hemifield collapsed across 80% valid and 50% valid conditions. The dots on the scalp map shows the positions of the EEG electrodes. The thicker dots on the posterior side of the scalp map shows the electrodes used for calculating CAS and CDA.

Figure 2a shows the contralateral alpha band (8–14 Hz) power suppression (i.e., the difference between the contralateral and ipsilateral power) with respect to the position of the cued item averaged across the electrode pairs of interest (P7/8, PO7/8, and O1/2). Cluster-based permutation tests showed significant contralateral alpha suppression in both the 80% valid condition (significant clusters: 338–830 ms, sum of t-values = 631.84, *p* < 0.001) and the 50% valid condition (significant clusters: 420–660 ms, sum of t-values = 337.80, *p* = 0.006), with initially no difference between the validity conditions. However, after about 700 ms from the onset of the retro-cue, the contralateral alpha suppression in 50% valid condition dropped back to the baseline, resulting in a significant difference between the 50% valid and 80% valid conditions in the later part of the delay period (significant clusters: 700–834 ms and 852–1000 ms; sum of t-values = 185.92, *p* = 0.013, and sum of t-values = 208.07, *p* = 0.003, respectively). These results suggest that early in the trial the cued item was attended independent of retro-cue reliability, but attentional prioritization was sustained for a longer period of time when the cue was more reliable.

**Figure 3:**
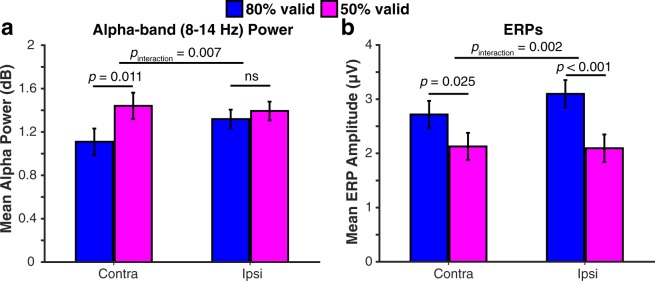
(**a**) Alpha power and (**b**) ERPs contralateral and ipsilateral to the side of the cued item averaged across the electrodes of interest during the time window in which cluster-based permutation testing revealed a significant reliability effect during the retention interval (i.e., 700–834 ms and 852–900 ms for alpha-band power and 400–514 for the CDA). 80% valid and 50% valid conditions are shown in different colors. The reliability effect was stronger at electrodes contralateral to the *non-cued* item for CDA, but contralateral to the *cued* item for alpha power. This is in line with the claim that the contralateral alpha suppression reflects the attentional selection of the cued item while the CDA reflects dropping of non-cued items. The error bars represent the standard error of the mean for the difference between 80% valid and 50% valid conditions separately for contralateral and ipsilateral values.

To look at whether retro-reliability affected contralateral and ipsilateral alpha-band power differently, we ran a repeated measures ANOVA with the factors of reliability (80% valid; 50% valid) and contralaterality (contra; ipsi) averaged across the time period that showed a significant reliability effect on contralateral alpha-band power as revealed by the cluster-based permutation testing described above. As seen in Fig. 3a, the retro-cue reliability affected the signal on the contralateral hemisphere relative to the cued item, *F*(1, 29) = 7.52, *p* = 0.010, η_p_^2^ = 0.21, 95% CI [0.08, 0.54], but not the ipsilateral hemisphere, *F*(1, 29) = 0.65, *p* = 0.426, η_p_^2^ = 0.02, 95% CI [−0.09, 0.23]. This was reflected in a significant laterality × reliability interaction, *F*(1, 29) = 8.27, *p* = 0.004, η_p_^2^ = 0.25. As described below, the CDA showed the opposite pattern where the reliability effect is stronger in the ipsilateral side than the contralateral side which is in line with a dissociation between the contralateral alpha suppression and the CDA.

#### Non-cued items were dropped from WM earlier and more often after highly reliable cues

Figure 2b shows the difference between contralateral and ipsilateral waveforms in respect to the location of the retro-cued item, averaged across the electrode pairs of interest (P7/8, PO7/8, and O1/2). Cluster-based permutation tests showed that for 80% valid blocks a significant contra-ipsi difference emerged early after the retro-cue and extended to the early part of our predefined CDA time window as well as the late part (significant clusters: 94–486 ms, sum of t-values = 1194.1, p < 0.001, and 534–1164 ms, sum of t-values = 1085.9, p < 0.001, after the retro-cue onset), while for 50% valid blocks there was a CDA only later in the trial, just before the test display (significant cluster: 690–1164 ms, sum of t-values = 813.38, p < 0.001). Interestingly, and in contrast to the alpha suppression, the difference between 80% valid and 50% valid conditions was mainly apparent early in the delay period (166–514 ms, sum of t-values = 579.20, p = 0.001), rather than late. These results suggest that non-cued items were dropped from WM right after the retro-cue after highly reliable cues but only later in the retention interval after less reliable cues.

In typical CDA studies participants store items presented laterally and the memory load mainly affects the signal contralateral to the memory items^67^. However, in the current study memory items are symetrical around fixation and retro-cue is presented laterally. If, as we propose, the CDA reflects dropping of the non-cued item, which is ipsilateral to the retro-cue, instead of a mnemonic or attentional boost for the cued item, which is contralateral to the retro-cue, then the retro-cue reliability should mainly modulate the ipsilateral vs. contralateral ERPs. To test this, we averaged the ERPs separately for contra- and ipsilateral to the cue across the time interval at which the condition difference was significant as revealed by the cluster-based permutation test As seen in Fig. 3b, the effect of retro-cue reliability was larger on the ipsilateral hemisphere, F(1, 29) = 15.52, *p* < 0.001, η_p_^2^ = 0.35, 95% CI [0.48, 1.52], compared to the contralateral hemisphere, F(1, 29) = 5.55, *p* = 0.025, η_p_^2^ = 0.16, 95% CI [0.08, 1.10], relative to the cued item. This difference was reflected in a significant laterality × reliability interaction, *F*(1, 29) = 11.24, *p* = 0.002, η_p_^2^ = 0.28. This result in in line with our claim that the emergence of the CDA mainly reflects dropping of the non-cued item (ipsilateral) as opposed to a mnemonic facilitation of the cued item (contralateral).

#### Correlations between EEG and behavior

**Figure 4:**
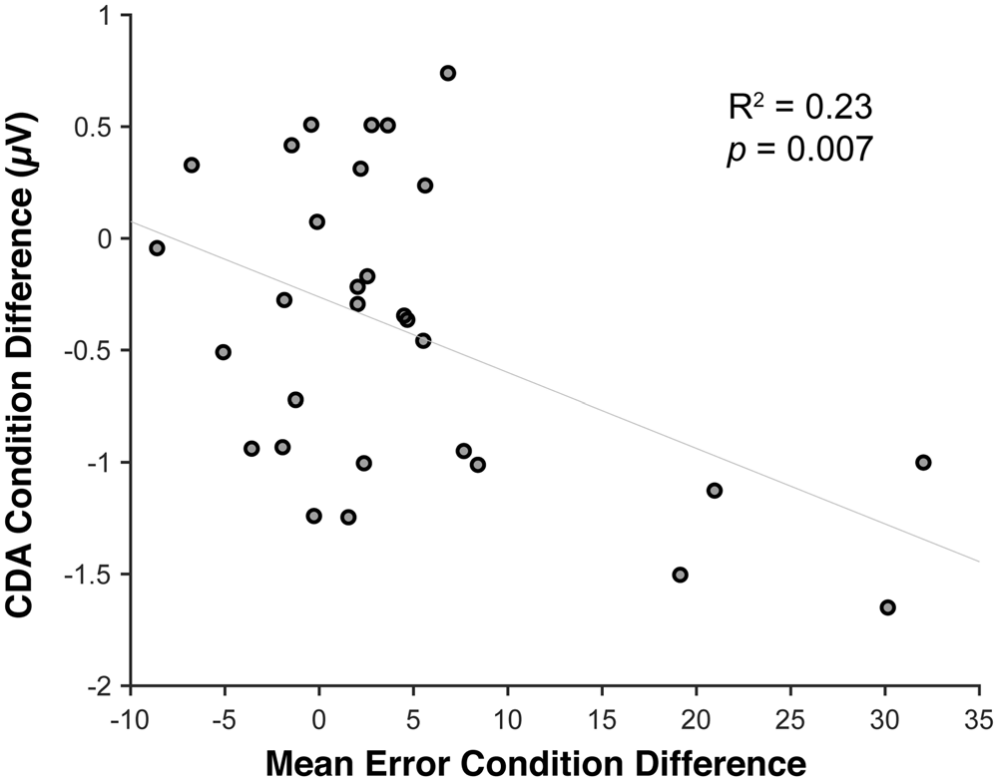
Correlation, across individuals, between reliability effects on CDA and invalid cueing cost. The x-axis shows the invalid cueing cost difference between reliability conditions (80% valid −50% valid) and the y-axis shows the CDA amplitude difference between reliability conditions (80% valid −50% valid). A larger CDA difference is correlated with a larger invalid cueing cost difference on behavior.

If CDA reflects dropping of non-cued items from working memory, then the CDA amplitude might predict behavioral costs of invalid retro-cues. To test if differences across reliability conditions in our EEG measures of interest predict behavior, we calculated the difference in invalid cueing cost (i.e. error in invalid trials minus error in valid trials) between 80% valid and 50% valid conditions, and correlated this with the CDA and contralateral alpha suppression differences between these two conditions. We calculated average CDA and contralateral alpha suppression at the time window in which the condition difference was significant within the retention interval as revealed by the aforementioned cluster-based permutation tests (400–514 ms for the CDA, and 700–834 ms and 852–900 ms the contralateral alpha suppression). There was no significant correlation between contralateral alpha suppression and behavior (*R*^2^ = 0.01, *p* = 0.68). However, as seen in Fig. 4, there was a significant correlation between CDA and behavior: Participants who had a larger invalidity cost difference between 80% valid vs. 50% valid conditions also had a larger CDA difference between these conditions, *R*^2^ = 0.23, *p* = 0.007. Note that although in the expected direction, with N = 30, and four participants having invalidity costs rather far from the mean, this correlation should be treated with some caution.

In addition, to test if variability in our EEG measures of interest predicts behavior, we computed at the level of individual data cells (participant; reliability condition; validity condition), the Pearson’s correlations between trial-level CDA and error, and between trial-level contralateral alpha power and error (except invalid trials of 80% reliable condition; as there were too few trials in this condition). There was no significant correlation (*p*s > 0.16).

#### Correlations between CDA and contralateral alpha suppression

To further test whether selective attention within WM relates to storage, we ran both across-participants and within-participants (i.e., across trials) correlation analyses between CDA and contralateral alpha suppression. There was no significant correlation between average CDA and average contralateral alpha suppression across participants for 80% valid blocks (*R*^2^ = 0.05, *p* = 0.21) or 50% valid blocks (*R*^2^ = 0.07, *p* = 0.17). Separate correlation analyses for time clusters that showed a significant effect of reliability on CDA and contralateral alpha supression also failed to reveal any significant correlation between the CDA and the contralateral alpha suppression (*R*^2^ < 0.03, *p*s > 0.33).

At a trial level, average Fisher transformed Pearson’s correlations between CDA and contralateral alpha suppression were not different from zero for 80% valid blocks, *t*(29) = 1.81, *p* = 0.080, 95% CI [−0.004, 0.068], or for 50% valid blocks, *t*(29) = −0.77, *p* = 0.449, 95% CI [−0.06, 0.02]. We should note that a correlation between contralateral alpha suppression and CDA, which is marginally significant, would have been expected in 80% valid condition because in this condition non-cued items are not only unattended but are also more likely to be dropped from WM. Our main hypothesis concerned a dissociation between selective attention and storage in WM when the fate of unattended items is less clear as in the 50% valid condition. We also tested the correlations against zero separately for the two time windows at which the reliability effect was significant for the CDA and contralateral alpha suppression. Again, no correlation was significnat (all *p*s > 0.259). Together, these results suggest that selective attention to one item did not predict storage of the other items in WM either on a trial level or on a participant level.

#### Ruling out the effects of eye position on EEG measures

In order to eliminate the effects of eye movements on the EEG signal, we removed trials with eye movements from analyses as revealed by HEOG. However, subtle but systematic eye movements toward the cued item can generate electrical potentials that can impact the EEG signal significantly. If the amount or magnitude of eye movements differ across reliability conditions, this can account for the differences in CDA and contralateral alpha suppression differences we observed between these two conditions. In order to evaluate this possibility, we calculated the difference in voltage between left and right HEOG electrodes and averaged it across the time window of interest (400–900 ms after the cue onset). The average HEOG value was 1.41 μV, which corresponds to saccades of less than only 0.09 visual degrees^66^. Moreover, although both were significantly different from zero (*p*s < 0.001), average HEOG values did not significantly differ between 80% valid blocks (*M* = 1.57 μV, *SEM* = 0.20) and 50% valid blocks (*M* = 1.26 μV, *SEM* = 0.21), *F*(1, 29) = 1.39, *p* = 0.248, η_p_^2^ = 0.05, 95% CI [−0.22, 0.84]. Thus, the magnitude of eye movements as measured with the HEOG was very small and did not differ across conditions. To further test if differences in eye movements across reliability conditions could have spuriously generated condition differences in our measures of interest, we calculated correlations across individuals (1) between the average contralateral minus ipsilateral HEOG and the average CDA, and (2) between the average contralateral minus ipsilateral HEOG and the average contralateral alpha suppression. Again, there was no significant correlation for either reliability condition (*R*^2^ = 0.10, *p* = 0.084, and *R*^2^ = 0.01, *p* = 0.587, respectively). Thus, we conclude that it is unlikely that eye movements spuriously generated the CDA and contralateral alpha suppression effects we observed in the present study.

## Discussion

Selective attention has been claimed to be essential for WM storage^12,14–16,18,19^. However, results regarding the costs of allocating attention away from WM representations have been conflicting^25,31,32,36,37,40^. To shed new light on these discrepant results, we used EEG indices of storage (i.e., CDA) and spatial selective attention (i.e., contralateral alpha suppression) within WM when the most task-relevant representation was cued. Critically, following our previous work that shows retro-cue costs are sensitive to the reliability of the cue, we manipulated the proportion of valid to invalid trials of the retro-cues across blocks (80% valid vs. 50% valid)^39^. We replicated our behavioral findings by showing that the effects of retro-cues on a continuous report recall task performance are larger for more reliable cues (i.e., 80% valid).

Importantly, here we show that these behavioral effects have a correlate in the amount of attention paid to the cued item on the one hand and the dropping of non-cued items on the other. Interestingly, we found that cue reliability mainly affected the timing and duration of different mechanisms, rather than their amplitude. Specifically, soon after the cue, contralateral alpha suppression was equal regardless of cue reliability, suggesting that the cue was used to direct *attention* to the cued representation as it presented useful information both when the cue was 50% valid or 80% valid. However, contralateral alpha suppression then persisted throughout the retention period only for highly reliable cues, while it quickly dropped to baseline for less reliable cues. This result indicates that attention was sustained on the cued item when participants could be reasonably sure that it would also be the tested item, while they also reverted to non-cued items when there was a decent probability of being tested on those too. As a measure of *storage* we took the CDA, which emerged early in the retention interval after highly reliable cues consistent with a rapid drop of the non-cued item from WM. Later in retention, a CDA also emerged for the low-reliability retro-cue condition, suggesting that eventually non-cued items were also dropped from WM after less reliable cues.

Thus, the time-resolved nature of our EEG measures reveals that the reliability of the retro-cue had dissociable effects on the dynamics of the CDA and contralateral alpha suppression. While for highly reliable cues non-cued items were both unattended and dropped from WM, for less reliable cues non-cued items were initially unattended but kept in WM. The latter result suggests that attentional selection of an item in WM is not directly accompanied by the loss of unattended items when there is a relatively high chance that these items could be relevant soon. Thus, our results support a dissociation between selective attention and storage in WM.

Non-cued items were unattended but kept in WM following less reliable retro-cues. To our knowledge, ours is the first study to observe simultaneous neural evidence for prioritization of attended items and active storage of unattended items in WM. This finding is in contrast with the claims that suggest WM storage is a direct reflection of selective attention in WM^14–16,18,19^, and supports the view that attentional prioritization of an item is a decision separate from dropping the remaining items^22–27^. This discrepancy between the two bodies of evidence is likely due to differences between the perceived future relevance of unattended items across studies since studies that observed costs for unattended items used highly reliable cues for which here we show that unattended items were dropped immediately.

Although unattended items in low-reliability cue blocks were initially kept in WM, they were eventually dropped from WM right before the onset of the test display. There are two explanations for the delayed loss of unattended items for less reliable cues. First, non-cued items might have become more vulnerable to interference from other items in WM due to being initially unattended following the retro-cue. Increased vulnerability in turn might have resulted in the deterioration of non-cued items through the retention interval. This scenario is in line with the evidence that proposes selective attention protects WM items against inter-item interference during storage^25^. Alternatively, non-cued items might have been stored in WM for a longer duration in an attempt to create passive memory traces for these items. Passive memory traces have been claimed to be employed for currently less relevant representations even at short intervals used in the present study^68–70^. Once their passive memory traces were established, non-cued items might have been deliberately dropped from WM in order to allocate all working memory resources to the most relevant item. This strategy would be effective given previous findings that show smaller perceptual interference for smaller memory loads in working memory^43–45^. Importantly, although both of these explanations support the protective role of selective attention for storage in WM, they do not go against our conclusion that selective attention and storage in WM are distinct constructs, as here we show that an item can be unattended but still actively stored in WM at a given time.

At first glance, our behavioral findings might seem to contradict our EEG findings: The CDA – taken as the WM storage index – by the time of the memory probe was equal for 80% valid condition and 50% valid condition. However, behavioral performance was worse for non-cued items in 80% valid condition. There are three explanations for this apparent discrepancy that are not mutually exclusive. First, non-cued items were dropped later in the retention interval for 50% valid condition compared to 80% valid condition. Thus, it is possible that lingering memory traces were stronger in 50% valid condition and participants were able to re-activate these traces when probed with a non-cued item at test. This proposal is consistent with studies that show items can be stored over short intervals through patterns of synaptic weights, without sustained neural activity^68,71–74^. Second, for less reliable cues participants might handover non-cued items to a passive memory store in anticipation of being probed with them. A voluntary hand-off from active WM to passive memory might take a longer time compared to simply dropping items from memory altogether, which would explain why the CDA emerges later in the 50% valid condition. This claim is consistent with studies that show currently less relevant items can be successfully reported even though their storage is not reflected in neural recordings^26,75^. Third, contralateral alpha suppression data suggests that the cued item was attended more in 80% valid vs. 50% valid condition. In other words, non-cued items were unattended more in 80% valid condition. Thus, it is possible that non-cued items suffered larger probe interference in 80% valid condition. This explanation is consistent with previous research that shows attention protects WM items from perceptual interference^43–45^. Importantly, here we show that unattended items can be stored actively in WM prior to any interference by the probe, as suggested by a dissociation between EEG indices of storage and attention in WM during the retention interval in 50% valid condition.

Selective attention to the cued item was sustained until the end of the trial for highly reliable cues, but not for less reliable cues. We propose that the allocation of attention back to non-cued items for less reliable cues reflects an attempt to revive previously unattended items that were being lost. This explanation is consistent with recent evidence that suggests weakly encoded representations, which are presumably also the weakly represented ones, are prioritized during WM retention in an attempt to prevent their loss^76^. Attentional reallocation to non-cued items was not observed for highly reliable cues. Given existing evidence that suggests the use of retro-cues is at least partly under strategic control^39,77,78^, we claim that an item that is being lost is attended only when it is relevant for the ongoing task. Thus, our results provide evidence for the flexible nature of WM by showing that selective attention can be strategically adjusted based on the perceived future relevance of WM items. In addition to its protective function, selective attention in WM has also been suggested to increase the accessibility of the attended item in a way that it effectively guides behavior in the external world^23,24,79,80^. Thus, we argue that the presence of an attentional prioritization mechanism within WM aids flexible behavior in a dynamic world where the relative importance of multiple relevant items required for the task at hand changes frequently.

Recently, retro-cue benefits have been claimed to reflect an increase in the accessibility of the cued item in the absence of sustained selective attention^81^. According to this idea, the cued item is first attended and selected in memory. Then, its status is reconfigured in a way to make it more accessible for behavior. After this reconfiguration is complete, sustained attention is *not* necessary to keep this item in a prioritized accessible state. This theoretical model is in line with the pattern of results in 50% valid condition of the present study where selective attention to the cued item was not sustained yet there were behavioral benefits for the cued item. It is thus possible that the cued item was reconfigured for accessibility without sustained attention. However, attention was sustained until the end of the trial in 80% valid blocks. Our results therefore show that, while a brief attentional selection might be sufficient for increasing the accessibility of a task-relevant item, highly relevant items are attended in a sustained manner.

The CDA has been traditionally defined as sustained relative negativity contralateral to the memory items presented on one hemifield of the screen while the other hemifield is ignored. Here, the memory display contained memory items on both hemifields. Thus, we hypothesized that the emergence of a CDA following a retro-cue would mean that the item contralateral to the retro-cue is continued to be stored while item ipsilateral to the retro-cue is dropped^57^. An alternative explanation for the emergence of the CDA is that it reflects a boost for the cued item instead of signaling the loss of the non-cued item^82^. Given that the CDA reflects storage of the items in the contralateral side^53,67^, an impact on the storage of the cued item should be reflected on the signal contralateral to the *cued item*. However, contrary to this alternative explanation, the retro-cue reliability mostly affected the signal contralateral to the *non-cued item*. This result suggests that the CDA in the present study was a result of dropping non-cued items instead of a mnemonic boost for the cued item^57,83^. On the other hand, the alpha power effect was specific to the contralateral side relative to the cued item instead of the ipsilateral side. This result is consistent with this signal reflecting attentional selection instead of distractor suppression^84^ (but see^85^).

It has been argued that the CDA and contralateral alpha suppression are strongly related signals, to the extent that the CDA is an output of an asymmetry in the amplitude of alpha-band oscillations^86^. This would mean that the CDA reflects the same *attentional selection* mechanism that contralateral alpha suppression does, instead of being an index *storage* per se. However, this conclusion was based on an experiment that manipulated neither WM load nor the task demands, thus confounding task relevance and storage. In the present study, by having multiple items with different task-relevance, we show that contralateral alpha suppression and CDA behaved differently across time. Moreover, we did not observe a correlation between the CDA and contralateral alpha suppression across participants (for a similar dissociation, see^51,87^) or within participants on a trial level. Lastly, the retro-cue reliability effect was reflected in differences in the signal ipsilateral to the cued item for the CDA, but contralateral to the cued item for the contralateral alpha suppression. Together, these results strongly suggest that CDA is not simply a reflection of lateral alpha power asymmetry.

The dissociable pattern of lateral alpha power asymmetry and CDA is consistent with previous studies that observed larger contralateral alpha suppression when a WM item was stored for a more demanding task while the CDA was unchanged^51,65^. Here we extend these findings by showing, across two conditions, both equal CDA and different contralateral alpha power, and also equal contralateral alpha power and different CDAs at different time points within the same dataset. Thus, our results provide strong evidence for a dissociation between these two signals. Together, these findings suggest that the CDA reflects storage in WM^56^ and the contralateral alpha suppression reflects the allocation of attention within WM^88^ and argue against a recent claim that suggests CDA reflects the current focus of attention instead of storage in WM^89^.

In sum, by manipulating the reliability of retro-cues that indicate which of multiple WM items is most likely to be tested, we show that unattended items were kept longer in WM, but only when there was a relatively high chance that they could later be tested. Thus, we propose that the decision to drop an item from WM is separate than the decision to allocate attention away from it and that these decisions can be flexibly adjusted based on dynamic changes in the relative importance of WM representations for the task at hand.

## Supplementary information

Supplementary Figure 1
