# Supplementary material for: EEG dynamics reveal a dissociation between storage and selective attention within working memory
Source: Sci Rep. 2019 Sep 18;9:13499. doi: 10.1038/s41598-019-49577-0 (PMC6751203; doi:10.1038/s41598-019-49577-0)
Supplement: Supplementary file 1 — Supplementary Figure 1 [file 41598_2019_49577_MOESM1_ESM.pdf]

Supplementary Figure 1.

for

EEG dynamics reveal a dissociation between storage and selective attention within working memory

Günseli, E.[1, 2]*, Fahrenfort, J.[2,3], van Moorselaar, D.[2,3], Daoultzis, K.[2,4], Meeter, M.[5], & Olivers, C. N. L.[2,3]

1. Columbia University, Department of Psychology, New York, USA
2. Vrije Universiteit Amsterdam, Department of Experimental and Applied Psychology, Amsterdam, Netherlands
3. Institute for Brain and Behavior Amsterdam, Amsterdam, Netherlands
4. Panteion University, Department of Psychology, Athens, Greece
5. Vrije Universiteit Amsterdam, LEARN!, Amsterdam, Netherlands

* Corresponding Author:
Eren Günseli
Columbia University
Gunseli.eren@gmail.com

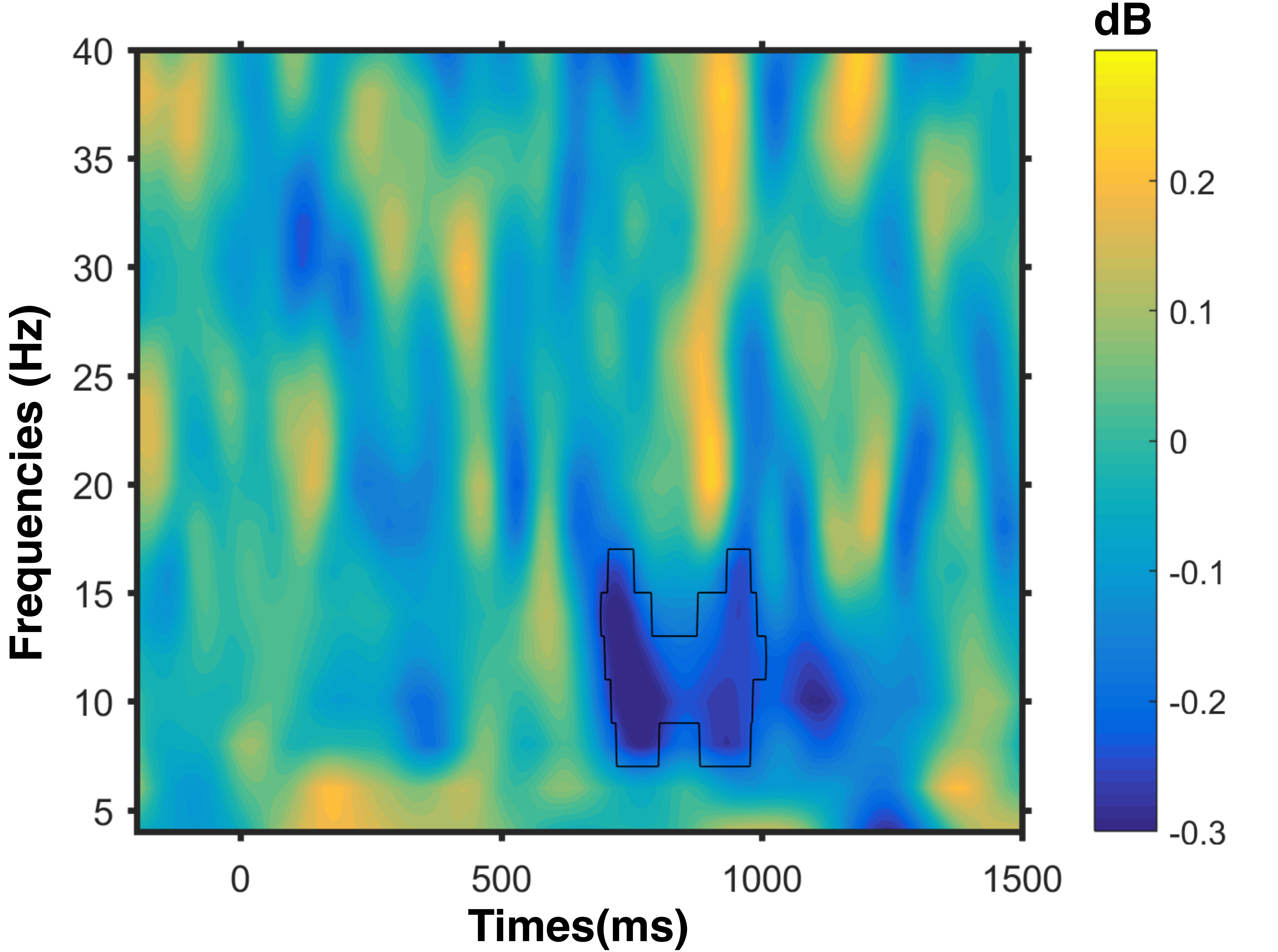
dB
40
35
30
25
20
15
10
5
Frequencies (Hz)
0
500
1000
1500
Times(ms)
0.2
0.1
0
-0.1
-0.2
-0.3
